# Cultural values and changes in happiness in 78 countries during the COVID-19 pandemic: An analysis of data from the World Happiness Reports

**DOI:** 10.3389/fpsyg.2023.1090340

**Published:** 2023-02-02

**Authors:** Ravi Philip Rajkumar

**Affiliations:** Department of Psychiatry, Jawaharlal Institute of Postgraduate Medical Education and Research (JIPMER), Puducherry, India

**Keywords:** happiness, culture, individualism–collectivism, power distance, long-term orientation, uncertainty avoidance, indulgence versus restraint, masculinity-femininity

## Abstract

The concept of happiness is consistent across cultures to a significant extent, and encompasses both internal (subjective) and external (situational) aspects. Cultural values and norms shape emotions and behavior from an early age, and hence play a key role in influencing cross-national variations in happiness. Cross-national variations in culture can thus play a key role in influencing the relationship between adverse circumstances, such as the COVID-19 pandemic, and happiness. The current study examines the relationship between the six dimensions of culture, defined by Hofstede and his colleagues, and subjective ratings of happiness in 78 countries, obtained before (2017–19) and during (2020–21) the COVID-19 pandemic, based on data from the most recent World Happiness reports. The key results were: (a) countries were as likely to experience an increase as a decrease in self-reported happiness during this period; (b) distinct domains of culture were significantly correlated with happiness at each time point, though there was a certain degree of overlap; (c) pre-pandemic levels of happiness were negatively associated with changes in happiness during the pandemic; and (d) among cultural dimensions, long-term orientation was positively associated with changes in subjective happiness, while indulgence was negatively associated with this variable. Certain cultural values may play an important part in fostering a path to well-being in the face of stressful or traumatic circumstances. This path may be similar to the concept of mature happiness, derived from existential philosophy, which is characterized by achieving a balance between the positive and negative aspects of one’s life.

## Introduction

Definitions of happiness vary across nations and cultures, but share certain core features. While happiness is commonly understood as satisfaction with one’s life, cross-national research has shown that happiness is a heterogeneous construct, incorporating both subjective, psychological dimension and broader social, relational, or contextual dimension (Uchida and Ogihara, 2012; Delle Fave et al., 2016; Cabanas and Gonzalez-Lamas, 2022). Other aspects of happiness, such as those relating to economic or social success, have been identified in empirical research (Doh and Chung, 2020); however, these two dimensions, which can be considered to reflect “inner harmony” and “social harmony,” appear to have primacy over the others. At the most fundamental, biological level, both aspects of happiness can be understood in terms of the molecular and neural mechanisms that regulate positive emotions and social behavior, and their evolutionary origins (Burgdorf and Panksepp, 2006; Niculescu et al., 2010). However, these lower-order factors are themselves shaped by cultural factors, which influence the degree of importance assigned to the subjective and contextual dimensions of happiness (Matsunaga et al., 2018). Some aspects of happiness appear to be similar in diverse cultural settings, even in childhood (Song et al., 2020), while others have been found to vary across cultures from the earliest stages of the life cycle (Rajhans et al., 2016; Liu et al., 2021). These variations are due to differences in parenting practices, and in the beliefs and rules governing both personal and social conduct and the evaluation of life events (Jordan and Graham, 2012; Simsek and Demir, 2014; Rudan et al., 2016; Reyes-Garcia et al., 2021; von Suchodoletz and Hepach, 2021; Wang, 2022). All of these are fundamentally “rooted in culture and tradition” (Daniels, 2019). Therefore, while the dimensions of happiness appear to be uniform across diverse cultures, culture can shape both the manner in which happiness is pursued (Ho et al., 2014) and the relationship between efforts to seek happiness and subjective well-being (Ford et al., 2015). A corollary of these observations is that as cultures change, these relationships are also modified (Timimi, 2010; Fuchsman, 2016).

The global COVID-19 pandemic has led to a deeper understanding of these concepts of happiness. As a global health crisis of unprecedented proportions, accompanied by widespread disruptions of social and economic life, this pandemic has been associated with elevated levels of subjective psychological distress (Cenat et al., 2021). In such a situation, one would logically expect a significant and widespread decrease in both subjective and situational happiness. However, this has not been the case. Surveys conducted among the general population in several countries, including Ecuador, Japan, Spain, and South Africa, found that a significant proportion of respondents reported average or even increased levels of happiness (Greyling et al., 2021; Gutierrez-Cobo et al., 2021; Kimura et al., 2022; Paz et al., 2022). Moreover, even in studies reporting a decrease in self-reported happiness, this change was modest; a study of over 8,000 Chinese adults found that mean happiness decreased by an average of 0.6% from the pre-pandemic period to the first year of the pandemic (Lin et al., 2021), while a study of respondents from 43 countries found that despite a slight decrease in average happiness, there were expectations of increased happiness in the near future (Muresan et al., 2022). This seemingly paradoxical finding can be explained if one considers that happiness is not a static but a dynamic phenomenon, and that mature happiness can be experienced even in the midst of adversity through a process of adaptation (Cloninger et al., 2012). The processes involved in this dynamic adaptation have been referred to by various terms, such as resilience, flourishing, salutogenesis, and post-traumatic growth; however, there is a significant degree of overlap between these constructs (Beckstein et al., 2022). More recently, existential positive psychology (PP2.0) has provided a framework within which these phenomena can be understood and applied at the psychological, social, and spiritual levels (Wong et al., 2021).

When studying the relationship between culture and happiness, it is important to distinguish between fine-grained, “micro”-level analyses, such as examinations of the relationship between parenting practices and subjective happiness in childhood, and broader, “macro”-level analyses (Ye et al., 2015). In the latter approach, cross-cultural variations in happiness are studied in terms of differences across one or more orthogonal dimensions identified through the analysis of large, multi-country data sets. One such approach that has been used in happiness studies is Hofstede’s six-factor model, in which a nation’s culture is described in terms of six dimensions: power distance, individualism–collectivism, masculinity-femininity, uncertainty avoidance, long-term orientation and indulgence vs. restraint (Hofstede et al., 2010). A detailed description of these cultural dimensions, and their potential relationships with happiness, is provided in Table 1 below.

**Table 1:** Hofstede’s six-factor model of culture and its relationship to happiness.

Factor	Definition	Scoring	Relationship with happiness
Power distance	The degree to which less powerful members of a society accept and expect inequality in power distribution.	Higher scores indicate a more “hierarchical” organization of society (e.g., Malaysia, with a score of 100) and lower scores indicate a more “egalitarian” society (e.g., Austria, with a score of 11)	High power distance has been negatively associated with subjective happiness in pre-pandemic research (Ye et al., 2015)
Individualism–collectivism	The degree to which society accords relative privilege to the individual or the wider social group / community	Higher scores indicate greater individualism (e.g., the United States, with a score of 91) and lower scores indicate collectivist values (e.g., Guatemala, with a score of 6).	Individualism may be associated with reductions in the interpersonal dimension of happiness (Ogihara and Uchida, 2014)
Masculinity-femininity	A social preference for either achievement, assertiveness, and competitiveness (masculinity) or care, nurturing and cooperation (femininity)	Higher scores indicate more masculine values (e.g., Slovakia, with a score of 100), and lower scores indicate more feminine values (e.g., Sweden, with a score of 5).	No significant associations between this dimension and happiness have been reported to date (Ye et al., 2015)
Uncertainty avoidance	The degree to which members of a society are comfortable with uncertainty and ambiguous situations	High scores indicate less comfort with ambiguity and a greater need for certainty and clarity (e.g., Greece, with a score of 100), and lower scores indicate a better ability to improvise in ambiguous situations (e.g., Singapore, with a score of 8)	High Uncertainty Avoidance may be associated with increased levels of unhappiness in relation to social change (Hofstede et al., 2010); however, an analysis of cross-national data found a positive association between this dimension and happiness (Ye et al., 2015)
Long-term orientation	Indicates a preference for pragmatism, modernity, perseverance and delayed gratification (future orientation), as opposed to traditionalism and resistance to change	Higher scores indicate a greater “future” orientation and pragmatism (e.g., the Republic of Korea, with a score of 100), while lower scores indicate a “past” orientation (e.g., Ghana, with a score of 4).	Higher Long-Term Orientation may moderate the association between economic status and happiness (Graafland, 2020)
Indulgence-restraint	The extent to which a society allows gratification of human drives related to pleasure or enjoyment	Higher scores indicate greater freedom to gratify desires for pleasure (e.g., Venezuela, with a score of 100), while lower scores indicate strict social norms and social disapproval of such desires (e.g., Pakistan, with a score of 0).	Indulgence has been positively associated with subjective happiness (Li et al., 2022) and prosocial behavior (Guo et al., 2018)

The psychological processes involved in adaptation to crises, and to the maintenance of happiness in the face of adversity, mechanisms are strongly influenced by cultural values and attitudes (Lawley et al., 2019; Mayer and Oosthuizen, 2020). For example, cultural collectivism has been associated with increased resilience following bereavement (Ariapooran et al., 2018). However, certain aspects of adaptation to adversity appear to be independent of culture (Mana et al., 2021), and it is not known which specific cultural dimensions contribute to happiness in the face of a global crisis.

Changes in happiness during a crisis such as the COVID-19 pandemic are also influenced by other demographic and psychological variables. These include economic development, social support, and a prior history of psychiatric illnesses such as depression and anxiety disorders (Osawa et al., 2022; Shams and Kadow, 2022). Economic factors may also indirectly affect happiness through their influence on the severity of the COVID-19 pandemic in a given country or region (Chang et al., 2022). An increase in the number of deaths due to COVID-19 is also associated with a consistent decrease in population levels of happiness over time (Greyling et al., 2021). Therefore, analyses of the relationship between culture and happiness over the course of the pandemic should be corrected for these potential confounding factors.

The aim of the current study was to examine whether national scores on each of Hofstede’s cultural dimensions were associated with changes in national levels of happiness during the COVID-19 pandemic, while correcting for the aforementioned confounders.

## Methods

The current study was a cross-national, ecological analysis of the relationship between Hofstede’s six dimensions of culture and levels of happiness at the national level, before and after the onset of the COVID-19 pandemic. Both cross-sectional and longitudinal analyses were performed.

### Data sources

#### Happiness

Data on estimated national levels of happiness were obtained from the World Happiness Report for the year 2021. The World Happiness Reports, which have been published annually from the year 2012 onward, are compiled by a panel of independent experts. These reports provide rankings of happiness for over 90 countries around the world based on a wide range of data, particularly the Gallup World Polls which collect data on subjective happiness and life satisfaction from each country (Helliwell et al., 2021). The 2021 report was selected because it provided composite indices of average national ratings of happiness for both the pre-COVID period (2017–2019) and the period following the onset of the COVID-19 pandemic (2020–2021) for a total of 95 countries. The happiness scores for each country range from 0 to 10, with higher scores indicating greater levels of self-reported happiness.

#### Cultural dimensions

Data on Hofstede’s dimensions of culture was obtained from the Hofstede Insights database, which provides scores on each of Hofstede’s six cultural dimensions for a total of 115 countries (Hofstede Insights, 2022). Each cultural dimension is assigned a score from 0 to 100, with lower or higher scores indicating a cultural orientation toward a particular “pole.” A description of these scores is provided in Table 1. For example, for the dimension “masculinity-femininity,” higher scores indicate a more masculine cultural orientation (characterized by an emphasis on achievement), and lower scores indicate a more feminine orientation (characterized by an emphasis on care and nurturing). Of the 115 countries covered by this database, 78 were also included in the World Happiness Report for 2021. These 78 countries were included in the current study.

#### Confounding factors

*I*n order to ensure that any observed associations between cultural values and happiness were not incidental, all analyses were corrected for certain confounding factors. The first of these the number of deaths related to the COVID-19 pandemic in each country, as measured by the estimated crude mortality rate and case-fatality ratio. Information on these variables was obtained as of March 20, 2021 (the date of the publication of the World Happiness Report) from the Johns Hopkins Coronavirus Resource Center (Johns Hopkins University of Medicine, 2022). The second was the general level of socioeconomic development achieved by each country as of 2019, prior to the onset of the COVID-19 pandemic. This was estimated using the Human Development Index, a composite measure of education, income, and life expectancy, obtained from the United Nations’ Human Development Report for the year 2019 (United Nations Development Programme, 2019). The third was the estimated prevalence of common mental disorders (depression and anxiety disorders) in each country for the year 2019. This variable was selected in view of the negative correlation between these disorders and self-reported happiness observed by earlier researchers (Keyes, 2005), as well as the finding that those with pre-existing mental disorders are more likely to experience psychological distress during the pandemic (Millroth and Frey, 2021). Data on this variable was obtained from the Global Burden of Disease Study 2019 (Institute for Health Metrics and Evaluation, 2022).

### Data analyses

Study variables were tested for normality using the Shapiro–Wilk test. As the COVID-19 mortality indices (crude mortality rate and case fatality rate) did not follow a normal distribution (*p* < 0.01, Shapiro–Wilk test), these variables underwent a natural logarithmic transformation prior to further analyses.

#### Cross-sectional analyses

Associations between each of the six cultural dimensions of Hofstede’s model and average happiness scores for each country were computed using Pearson’s correlation coefficient (*r*) for the pre-pandemic (2017–19) and pandemic (2020–21) periods. Correlation coefficients between happiness scores and potential confounding factors (COVID-19 mortality indices, Human Development Index, and prevalence of common mental disorders) were also computed. Based on these results, partial correlation analyses were then carried out to examine if any of the relationships between culture and happiness remained significant after correcting for confounders significantly associated with either variable. The strength of each correlation was quantified using standard guidelines for psychological research as follows: absolute value of *r* (|*r*|) *=* 0.1–0.39, weak correlation, |*r*| = 0.4–0.69, moderate correlation, and |*r*| = 0.7–0.99, strong correlation (Akoglu, 2018).

#### Longitudinal analyses

*T*he paired samples *t*-test was used to examine whether there was a significant change in happiness scores across countries between the periods 2017–19 and 2020–21. Countries were then categorized according to whether their happiness score had increased or decreased during the COVID-19 pandemic, and the percentage change in happiness score was computed for each country. Mean differences in baseline cultural dimensions and in confounding variables between these two groups of countries were examined using the independent samples *t*-test. A cross-lagged regression analysis was carried out to examine whether the relationship between culture and happiness was suggestive of a causal relationship. This possibility was further explored using an analysis of covariance (ANCOVA) using any confounding factors that differed significantly between groups as covariates. Finally, the correlations between changes in happiness scores and cultural dimensions were examined using Pearson’s *r* (unadjusted and adjusted for confounders).

All tests were two-tailed, and a significance level of *p* < 0.05 was used for all analyses.

## Results

A total of 78 countries were included in the current analysis, including 38 countries from Europe, 16 from the Asia-Pacific region, 13 from the American continent, and 11 from Africa. Mean happiness scores, given as mean (standard deviation), were 5.91 (1.05) in 2017–19, with a maximum of 7.81 (Finland) and a minimum of 3.48 (Tanzania). In 2020–21, the corresponding value was 5.94 (0.96), with a maximum of 7.89 and a minimum of 3.79 in the same countries, respectively. Happiness scores at both time points were very strongly correlated with each other (*r* = 0.94, *p* < 0.001).

### Cross-sectional associations between cultural dimensions and happiness scores

Results of the correlations between happiness scores and Hofstede’s cultural dimensions, as well as between these scores and potential confounding variables, are presented in Table 2. It can be seen that at both time points, happiness scores were positively correlated with scores on the cultural dimensions of Individualism–Collectivism and Indulgence-Restraint. In other words, higher individualism and higher indulgence were associated with higher happiness scores. On the other hand, scores on the cultural dimension of Power Distance were negatively correlated with happiness scores at both periods, suggesting that high Power Distance was negatively associated with happiness. For the period 2020–21 alone, corresponding to the COVID-19 pandemic, the cultural dimension of Long-Term Orientation was positively correlated with happiness scores, though the strength of this correlation was weak (*r* = 0.27).

**Table 2:** Correlation matrix of associations between national happiness scores, Hofstede’s cultural dimensions, and potential confounding before and during the COVID-19 pandemic.

Variable	1 H17	2 H20	3 PD	4 IC	5 MF	6 UA	7 LTO	8 IVR	9 HDI	10 ANX	11 DEP	12 CMR	13 CFR
1	–	0.94^†^	−0.60^†^	0.60^†^	−0.12	−0.09	0.13	0.45^†^	0.82^†^	0.47^†^	−0.11	0.24^*^	−0.31^†^
2		–	−0.63^†^	0.66^†^	−0.16	−0.13	0.27^*^	0.31^†^	0.84^†^	0.39^†^	−0.08	0.20	−0.33^†^
3			–	−0.74^†^	0.20	0.40^†^	0.06	−0.38^†^	−0.57^†^	−0.46^†^	−0.23^*^	−0.01	0.32^†^
4				–	−0.02	−0.38^†^	0.13	0.21	0.65^†^	0.40^†^	0.21	0.16	−0.21
5					–	0.02	0.01	0.04	−0.12	−0.11	−0.18	−0.05	0.24^*^
6						–	0.18	−0.25^*^	0.04	−0.08	−0.27^*^	0.40^†^	0.18
7							–	−0.49^†^	0.36^†^	−0.24^*^	−0.32^†^	0.19	0.06
8								–	0.15	0.34^†^	0.12	−0.12	−0.15
9									–	0.43^†^	−0.14	0.37^†^	−0.26^*^
10										–	0.35^†^	0.15	−0.09
11											–	−0.15	−0.10
12												–	0.26^*^

H17, World Happiness Report score (2017–18); H20, World Happiness Report score (2020–2021); PD, Power Distance; IC, Individualism–Collectivism; MF, Masculinity-Femininity; UA, Uncertainty Avoidance; LTO, Long-Term Orientation; IVR, Indulgence Versus Restraints; HDI, Human Development Index (2017); ANX, prevalence of anxiety disorders (Global Burden of Disease Study, 2017); DEP, prevalence of depression (Global Burden of Disease Study, 2017); CMR, COVID-19 crude mortality rate; CFR, COVID-19 case fatality ratio. ^*^Significant at *p* < 0.05; ^†^Significant at *p* < 0.01.

When examining confounding variables, the Human Development Index and the prevalence of anxiety disorders were positively correlated with happiness scores; the former correlation (*r* = 0.82 to 0.84) was strong, while the latter was moderate (*r* = 0.39 to.47). During the pandemic, COVID-19 case fatality rate was negatively correlated with happiness scores. There was no observed correlation between COVID-19 crude mortality rates and happiness.

### Partial correlation analyses

Given the positive associations of happiness scores with the Human Development Index and the prevalence of anxiety disorders, partial correlation analyses of the relationships between happiness scores and cultural dimensions were carried out holding these two factors constant. In these analyses, the happiness score in 2017–18 was negatively correlated with Power Distance (partial *r* = −0.24, *p* = 0.039) and Long-Term Orientation (partial *r* = −0.25, *p* = 0.035) and positively correlated with Indulgence versus Restraint (partial *r* = 0.55, *p* < 0.001). The happiness score in 2021 was negatively correlated with Power Distance (partial *r* = −0.34, *p* = 0.003) and Uncertainty Avoidance (partial *r* = −0.29, *p* = 0.011) and positively correlated with Individualism–Collectivism (*r* = 0.27, *p* = 0.017) and Indulgence versus Restraint (*r* = 0.33, *p* = 0.005). In other words, the associations between two specific cultural dimensions (Power Distance and Indulgence versus Restraint) and national happiness were consistent over time and retained significance even after adjustment for confounders.

### Changes in happiness during the pandemic

When comparing mean happiness scores from 2017–19 to 2020–21, it was found that there was no significant difference in this variable across time points (paired-samples *t* = −0.87, *p* = 0.388, *df* = 77). Though there was a slight increase in the mean happiness score, this was modest in magnitude (Cohen’s *d* = 0.1) and not statistically significant.

When comparing happiness before and during the pandemic, it was found that an equal number of countries (*n* = 39 in each case) showed an increase or a decrease in happiness scores. The mean percentage change in happiness scores was 1.22 ± 7.36%, with a maximum decrease of-15.42% seen in the Philippines and a maximum increase of 28.7% observed in Zambia.

When these countries were compared in terms of baseline (pre-pandemic) characteristics, it was found that countries with an increase in happiness had significantly higher scores on Long-Term Orientation (*t* = 2.28, *p* = 0.025) and lower scores on Indulgence versus Restraint (*t* = −4.3, *p* < 0.001). Among confounding factors, only the pre-pandemic prevalence of anxiety was significantly lower in countries reporting an increase in happiness (*t* = −2.51, *p* = 0.014). It was also observed that countries with a decrease in happiness scores over this period had significantly higher pre-pandemic happiness scores (*t =* 3.24, *p* = 0.002).

### Longitudinal associations between cultural dimensions and changes in happiness during the pandemic

Three methods were adopted to test the hypothesis of a relationship between Hofstede’s cultural dimensions and changes in happiness during the COVID-19 pandemic. The first method adopted was a cross-lagged regression analysis, the results of which are presented in Table 3. From this analysis, it can be seen that only the cultural dimension of Long-Term Orientation showed a possible causal relationship with happiness scores, as indicated by a positive prospective correlation but no significant correlation in the opposite direction.

**Table 3:** Cross-lagged regression analysis of the relationship between Hofstede’s cultural dimensions and national happiness scores before and during the COVID-19 pandemic.

Cultural dimension	Correlation with 2017–18 happiness score	Correlation with 2021–21 happiness score	Inference
Power distance	−0.60 (<0.001)	−0.63 (<0.001)	No causal relationship
Individualism–collectivism	0.60 (<0.001)	0.66 (<0.001)	No causal relationship
Masculinity-femininity	−0.12 (0.280)	−0.16 (0.160)	No causal relationship
Uncertainty avoidance	−0.09 (0.417)	−0.13 (0.273)	No causal relationship
Long-term orientation	**0.13 (0.291)**	**0.27 (0.023)**	**Possible causal relationship**
Indulgence vs. restraint	0.45 (<0.001)	0.31 (0.008)	No causal relationship

All correlation coefficients are given in the form: Pearson’s *r* (*p* value). Values in **bold** indicate a possible causal relationship (i.e., a significant correlation between pre-pandemic cultural scores and pandemic happiness scores, but not the converse).

The second method was a bivariate correlation analysis of the relationship between cultural dimension scores and the percentage of change in happiness scores during the pandemic. The results of these correlations are presented in Table 4. In these analyses, Long-Term Orientation was positively correlated with changes in happiness (*r* = 0.27*, p* = 0.02), while Indulgence vs. Restraint was negatively correlated with changes in happiness (*r* = −0.44, *p* < 0.001). The association between Indulgence vs. Restraint and changes in happiness remained significant when controlling for Long-Term Orientation (partial *r* = −0.37*, p* = 0.002), but the converse was not true (partial *r* = 0.07, *p* = 0.566). When adjusting for the possible confounding effects of the prevalence of anxiety disorders before the pandemic, the association with Indulgence versus Restraint remained significant (*r* = −0.36, *p* = 0.002), but the association with Long-Term Orientation was reduced to a trend (*r* = 0.21, *p* = 0.072).

**Table 4:** Bivariate correlations between Hofstede’s cultural dimensions and the percentage of change in national happiness scores from 2017–18 to 2020–21.

Cultural dimension	Power distance	Individualism–collectivism	Masculinity-femininity	Uncertainty avoidance	Long-term orientation	Indulgence vs. restraint
Correlation with change in happiness scores	0.10 (0.398)	−0.04 (0.739)	−0.06 (0.597)	−0.10 (0.410)	0.27 (0.020)^*^	−0.44 (<0.001)^†^
Correlation with change in happiness scores, adjusted for baseline prevalence of anxiety disorders	−0.06 (0.608)	0.10 (0.379)	−0.10 (0.369)	−0.13 (0.271)	0.21 (0.072)	−0.36 (0.002)^†^

All correlation coefficients are presented as Pearson’s r or partial r (value of p). *Significant at *p* < 0.05; †Significant at *p* < 0.01.

The third and final method was an analysis of covariance (ANCOVA) using Indulgence vs. Restraint and Long-Term Orientation as the dependent variables and the percentage of change in national happiness scores as the independent variable. The results of these analyses are presented in Table 5. When Indulgence vs. Restraint was taken as the dependent variable, it remained significantly different across groups, and the association with Long-Term Orientation was also found to be significant. When Long-Term Orientation was taken as the dependent variable, it was not significantly different across groups, though a meaningful effect of Indulgence vs. Restraint was identified. The prevalence of anxiety disorders was not significantly associated with between-group differences in either model.

**Table 5:** Analyses of covariance of the association between Hofstede’s cultural dimensions and changes in happiness scores during the COVID-19 pandemic.

Independent variable	Covariates	Result for independent variable	Result for covariates
Indulgence vs. restraint	LTO, Anxiety	*F =* 9.44, *p* = 0.003**	LTO: *F =* 13.40, *p* < 0.001** Anxiety: *F* = 2.15, *p* = 0.147
Long-term orientation	IVR, Anxiety	*F* = 0.03, *p* = 0.869	IVR: *F* = 13.40, *p* < 0.001** Anxiety: *F* = 0.53, *p* = 0.468

Countries were compared based on whether happiness scores increased or decreased during the pandemic. Abbreviations: Anxiety, estimated prevalence of anxiety disorders (%) as per the Global Burden of Disease Study, 2019; IVR, Indulgence vs Restraint; LTO, Long-Term Orientation. * Significant at *p* < 0.05; ** Significant at *p* < 0.01.

## Discussion

In the early stages of the COVID-19 pandemic, concerns were raised that this unprecedented crisis would lead to a rapid increase in mental ill-health and a decrease in subjective happiness (Rose et al., 2020). These concerns appeared to be corroborated by reports during this time period. For example, a survey of Chinese adults found that the onset of the pandemic was associated with a decrease in subjective happiness of over 70% (Yang and Ma, 2020), while a similar survey of adults in Spain found that 44% of respondents reported a decrease in feelings of optimism and positivity (Hidalgo et al., 2020). However, even at this stage, some researchers felt that such concerns were overstated, and that even if an increase in distress or unhappiness was observed, it was likely to vary markedly across populations, and to reflect the combined influence of baseline social and economic factors alongside pandemic-related factors (Rose et al., 2020). The subsequent course of events has tended to support the latter view: deteriorations in mental health status have been far from uniform (Shevlin et al., 2021), and increases in life satisfaction and happiness have been reported from diverse geographical regions, particularly in the later stages of the pandemic (Greyling et al., 2021; Gutierrez-Cobo et al., 2021; Henseke et al., 2022; Kimura et al., 2022; Paz et al., 2022). Moreover, it was observed that in certain settings, individuals came to value the interpersonal or relational dimension of happiness to a greater extent than they did pre-pandemic (Bimonte et al., 2022).

Both the experience of happiness, and its relationship to adversity, are crucially shaped by cultural values. Though various definitions of culture have been proposed, Hofstede has conceptualized cultural values as “software of the mind” which are not biologically determined, but have evolved in response to environmental and human challenges in a historically contingent manner (Hofstede et al., 2010). In fact, there is evidence that past outbreaks of infectious disease may have influenced the development of specific cultural values: regions with a higher burden of such diseases may have “evolved” a more collectivist orientation in order to cope more effectively with them (Fincher et al., 2008; Shapouri, 2022). However, most research in this field has focused on Individualism–Collectivism and not on other dimensions of culture that may be equally or even more important in influencing the behavioral and psychological responses to a large-scale crisis.

In the current study, certain cultural dimensions (Power Distance, Individualism–Collectivism, and Indulgence-Restraint) were significantly associated with happiness scores for each country both pre-and post-pandemic. However, when examining changes in happiness in the course of the pandemic, the cultural dimensions most strongly associated with this variable were Indulgence-Restraint, and to a lesser extent, Long-Term Orientation. This suggests that the cultural factors associated with a baseline or “stable” level of happiness are not necessarily the same factors that influence the relationship between adversity and happiness. This supposition is corroborated by the findings that countries with a higher pre-pandemic happiness score were more likely to experience a decrease in happiness during the pandemic. In the case of Indulgence-Restraint, a paradoxical phenomenon was observed: this cultural dimension was positively correlated with total happiness scores, but negatively correlated with changes in happiness during the pandemic. In the case of Long-Term Orientation, a correlation with happiness scores was observed only during the pandemic, and this dimension was positively correlated with changes in happiness.

Prior research on these dimensions suggests that Indulgence-Restraint is positively correlated, and Long-Term Orientation negatively correlated, with prosocial behavior (Guo et al., 2018). Therefore, it is possible that these aspects of culture may have influenced happiness in the opposite direction during a period of widespread social distancing and isolation (Su et al., 2022). Long-Term Orientation has also been found to moderate the relationship between economic freedom and subjective well-being (Graafland, 2020). Thus, it is possible that the personal values associated with this dimension, such as patience, perseverance, and delaying gratification, may have enabled individuals in such cultures to cope better with the economic hardships caused by the pandemic (Richards et al., 2022). On the other hand, Indulgence represents a tendency toward gratification of human desires, “enjoying” life, and having “fun” (Hofstede et al., 2010; Smith, 2011). This would explain why this dimension is associated with happiness during times of relative normalcy. However, the thwarting of these tendencies by an event such as the COVID-19 pandemic could conceivably lead to a decrease in subjective happiness (Simon et al., 2022). However, this finding may not extend to the relational dimension of happiness: a global survey of over 9,900 parents found that Indulgence was negatively associated with parental burnout and unhappiness during the initial stages of the pandemic, with higher Indulgence predicting lower unhappiness (van Bakel et al., 2022).

In contrast to the findings relating cultural dimensions and happiness over the course of the pandemic, relatively few associations were found when examining possible confounding factors: the Human Development Index was associated with total happiness scores but not with changes in happiness, while the COVID-19 case-fatality ratio was negatively associated with total happiness scores. Among mental disorders, anxiety disorders, but not depression, were negatively associated with changes in happiness during the pandemic. The latter finding is unexpected, and a detailed exploration of its implications is beyond the scope of this paper. However, it has been observed that in cultures placing a high emphasis on the “pursuit of happiness” (i.e., high Indulgence), this “pursuit” may itself generate significant anxiety (Cloutier et al., 2020; Humphrey et al., 2021). Given that the prevalence of anxiety disorders was positively correlated with Indulgence, such findings may offer a possible explanation for this result.

Certain key limitations of this study should be borne in mind when interpreting its results. First, the findings are based on a particular model of culture and on estimates of specific parameters obtained from survey data, which necessarily involve a certain margin of error. Second, the World Happiness Report data provided information on the subjective dimension of happiness; therefore, it was not possible to examine the relationship between culture and other aspects of happiness. Third, due to the correlational nature of these results, firm conclusions regarding causality cannot be drawn. Fourth, it is possible that other confounding factors, such as economic inequality, religion, social support, spirituality, and even innate biological differences between populations, could account for some of the variation observed. Fifth, there was a relative under-representation of Asian and African countries in the study sample, which limits the extent to which these results can be generalized to non-Western cultures. Sixth, the period covered by the available data included only the first year of the pandemic: it is not known if these findings will remain significant over a longer period of time. Finally, these findings should not be taken as promoting the superiority of one culture over another. As mentioned earlier, cultural values represent historically contingent adaptations and compromises, and it is possible that the values identified as positively associated with happiness during the pandemic may have quite different consequences in other situations.

Nevertheless, the current study’s results are in line with the proposal for a “new behavioral economics of happiness.” Such a behavioral economics would extend beyond the pursuit of pleasure or subjective satisfaction, embrace the “hard questions” of dealing with suffering, and address not just the relational but the transcendental aspects of happiness (Wong et al., 2021). The COVID-19 pandemic has exposed certain hard truths about the limitations of pre-pandemic attitudes and beliefs. The cultural values that are consistent with the former, pre-pandemic model of happiness are not necessarily consistent with the latter. This has been demonstrated by a recent study of individuals from 30 different countries. In these individuals, a conventional model of happiness based on subjective and objective well-being did not protect against psychological distress during the pandemic, but a model based on mature happiness and adaptation to adversity was protective (Carreno et al., 2021). It is possible that a reduced emphasis on gratification of desires and subjective enjoyment, and a cultivation of the virtues associated with the cultural dimension of Long-Term Orientation, such as perseverance and the ability to delay gratification and look toward the future, could help in building and sustaining a more mature form of happiness among individuals and communities. It is also possible that, regardless of the cultural orientation of a given country, reliance on processes that transcend cultural variations could aid this process. These include a connection with Nature (Svoray et al., 2022) and the construction of a sense of meaning and purpose in the face of suffering (Mana et al., 2021).

## Conclusion

The results of the current study suggest that certain dimensions of a nation’s culture may influence their reported levels of happiness in the context of a global crisis. Though the findings of this study should be interpreted cautiously, they suggest that certain culturally determined values and patterns of behavior may influence a populations’ capacity to adapt to such a crisis. The identification of these patterns of thought and conduct may be of use in building resilience and fostering adaptation in such situations, and such approaches could be profitably combined with more general, culturally invariant strategies aimed at fostering mature happiness in communities.

## Data availability statement

The raw data supporting the conclusions of this article will be made available by the authors, without undue reservation.

## Author contributions

The author confirms being the sole contributor of this work and has approved it for publication.

## Conflict of interest

The author declares that the research was conducted in the absence of any commercial or financial relationships that could be construed as a potential conflict of interest.

## Publisher’s note

All claims expressed in this article are solely those of the authors and do not necessarily represent those of their affiliated organizations, or those of the publisher, the editors and the reviewers. Any product that may be evaluated in this article, or claim that may be made by its manufacturer, is not guaranteed or endorsed by the publisher.
