# Supplementary material for: Cultural values and changes in happiness in 78 countries during the COVID-19 pandemic: An analysis of data from the World Happiness Reports
Source: Front Psychol. 2023 Feb 2;14:1090340. doi: 10.3389/fpsyg.2023.1090340 (PMC9932538; doi:10.3389/fpsyg.2023.1090340)
Supplement: Supplementary file 1 [file Table_1.DOCX]

**Supplementary Table 2: Complete correlation matrix for all study variables**

| **Variable** | **H2019** | **H2021** | **PD** | **IC** | **MF** | **UA** | **LTO** | **IVR** | **C19-Prev** | **C19-CMR** | **C19-CFR** | **HDI** | **Dep** | **Anx** |
| --- | --- | --- | --- | --- | --- | --- | --- | --- | --- | --- | --- | --- | --- | --- |
| **H2019** | - | .94** (<.001) | -.60** (<.001) | .60** (<.001) | -.12 (.280) | -.09 (.417) | .13  (.291) | .45** (<.001) | .36** (<.001) | .24* (.038) | -.31** (.006) | .82** (<.001) | -.11 (.347) | .47** (<.001) |
| **H2021** |  | - | -.63** (<.001) | .66** (<.001) | -.16 (.160) | -.13  (.273) | .27* (.023) | .31** (.008) | .32** (.004) | .20 (.087) | -.33** (.004) | .84** (<.001) | -.08 (.497) | .39** (<.001) |
| **PD** |  |  | - | -.74** (<.001) | .20 (.080) | .40** (<.001) | .06 (.617) | -.38** (.001) | -.12 (.298) | -.01  (.989) | .32** (.005) | -.56** (<.001) | -.23* (.046) | -.46** (<.001) |
| **IC** |  |  |  | - | -.02 (.838) | -.38** (<.001) | .13 (.276) | .21** (.084) | .24* (.035) | .16 (.174) | -.21 (.068) | .65** (<.001) | .21 (.062) | .40** (<.001) |
| **MF** |  |  |  |  | - | .02 (.890) | .01 (.940) | .04 (.746) | -.14 (.231) | -.05 (.644) | .242* (.035) | -.12 (.306) | -.18 (.113) | -.11 (.318) |
| **UA** |  |  |  |  |  | - | .18 (.121) | -.25* (.036) | .34** (.002) | .40** (<.001) | .18 (.119) | .04 (.714) | -.27* (.019) | -.08 (.491) |
| **LTO** |  |  |  |  |  |  | - | -.49** (<.001) | .17 (.152) | .19 (.113) | .06 (.604) | .36** (.002) | -.32** (.005) | -.23* (.046) |
| **IVR** |  |  |  |  |  |  |  | - | -.07 (.564) | -.12 (.332) | -.15 (.213) | .15 (.221) | .12 (.324) | .342** (.003) |
| **C19-Prev** |  |  |  |  |  |  |  |  | - | .94** (<.001) | -.09 (.423) | .48** (<.001) | -.11 (.337) | .19 (.110) |
| **C19-CMR** |  |  |  |  |  |  |  |  |  | - | .26* (.026) | .37** (.001) | -.15 (.212) | .15 (.203) |
| **C19-CFR** |  |  |  |  |  |  |  |  |  |  | - | -.26* (.024) | -.10 (.373) | -.09 (.458) |
| **HDI** |  |  |  |  |  |  |  |  |  |  |  | - | -.14 (.221) | .43** (<.001) |
| **Dep** |  |  |  |  |  |  |  |  |  |  |  |  | - | .35** (.002) |

Abbreviations: Anx, estimated prevalence of anxiety disorders; C19-Prev, estimated prevalence of COVID-19; C19-CMR, crude mortality rate due to COVID-19; C19-CFR, case-fatality ratio for COVID-19; Dep; estimated prevalence of depression; H2019, happiness score for the period 2017-2019; H2021, happiness score for the period 2020-2021. HDI, Human Development Index; IC, Individualism-Collectivism; IVR, Indulgence Versus Restraint; LTO, Long-Term Orientation; MF, Masculinity-Femininity; PD, Power Distance; UA, Uncertainty Avoidance. See the text for details of the sources for each variable.

All correlations are given as Pearson’s *r* (*p-*value)

* Significant at *p* < .05.

** Significant at *p* < .01.
